# *ITGB5* mutation discovered in a Chinese family with blepharophimosis-ptosis-epicanthus inversus syndrome

**DOI:** 10.1515/biol-2021-0129

**Published:** 2021-12-10

**Authors:** Tianling Cheng, Xiaobin Yuan, Shaopeng Yuan, Jianying Zhu, Shengjian Tang, Yujie Zhang

**Affiliations:** Institute of Plastic Surgery, Weifang Medical University, Weifang, Shandong, 261000, China; Department of Disease Control and Prevention, Qingdao Hospital of Traditional Chinese Medicine, Qingdao, Shandong, 266000, China; Department of Research and Development, Beijing Shunlei Technology Co., Ltd, Beijing, 100000, China; Zibo Yimei Plastic Surgery Hospital, Zibo, Shandong, 255000, China

**Keywords:** BPES, *ITGB5*, whole-exome sequencing, pathogenic genes, dominant inheritance

## Abstract

Blepharophimosis-ptosis-epicanthus inversus syndrome (BPES) is a rare autosomal-dominant genetic disorder, and mutations in the forkhead box L2 (*FOXL2*) gene are one of the major genetic causes. As this study shows, there are many patients with BPES who do not have *FOXL2* mutations, as the screening results in all family members were negative. Using whole-exome sequence analysis, we discovered another possible mutational cause of BPES in integrin subunit beta 5 (*ITGB5*). The *ITGB5* mutation (c.608T>C, p.Ile203Thr) appears in the base sequence of all BPES^+^ patients in this family, and it appears to be a three-generation-inherited mutation. It can cause changes in base sequence and protein function, and there may be cosegregation of disease phenotypes. *ITGB5* is located on the long arm of chromosome three (3q21.2) and is close to the known pathogenic gene *FOXL2* (3q23). This study is the first to report *ITGB5* mutations in BPES, and we speculate that it may be directly involved in the pathogenesis of BPES or indirectly through the regulation of *FOXL2*.

## 1 Introduction

Blepharophimosis-ptosis-epicanthus inversus syndrome (BPES) is a rare congenital malformation, most of which is inherited in an autosomal-dominant pattern, with a reported incidence of 1:50,000 [1,2]. BPES patients usually have common facial features, such as ptosis, narrow fissure, inverted epicanthus, widened epicanthus spacing, and low bridge of the nose, and also can be accompanied by microcephaly, premature ovarian failure, growth retardation, hypopituitary function, low intelligence, and other congenital abnormalities [3,4,5,6]. In addition, some BPES patients may also have small eyes, nystagmus, eyelid varus or ectropion, strabismus, and lacrimal system anomalies, which can affect the visual development [7]. Clinically, BPES is divided into types I and II. Female patients with type I are usually associated with infertility, primary amenorrhea or early menopause, and atrophy of a smaller uterus and ovaries. The main difference from type I is both male and female patients with type II can have children. BPES affects the appearance and ovarian function and fertility in female patients, which is a severe threat to physical and mental health [8].

Exons are the coding regions of proteins and, therefore, the most genetically valuable regions of DNA. All exons in the genome are termed the exome. The human genome is about 1.8 × 10^5^ exons, accounting for only 1% of the human genome. Studies have shown that base mutations cause the occurrence and development of a variety of diseases in exons [9,10,11]. Whole-exome sequencing (WES) utilizes sequence capture technology to capture and enrich exome DNA and then performs high-throughput sequencing. When studying diseases caused by genetic mutations, WES has obvious advantages. Compared with other genetic detection technologies, WES has the advantages of full detection range, high read depth, and reasonable cost [12]. Therefore, WES technology is currently considered to be the most efficient and cost-effective genetic testing technology.

*FOXL2* is known to be the major pathogenic gene in BPES [13]. Using a combination of methods to detect mutations, the underlying genetic defect can be identified in the majority (88%) of patients with typical BPES. Overall, *FOXL2* mutations accounted for 81% of the genetic defects found in BPES [14,15]. In this study, *FOXL2* mutation screening and WES were performed on a single Chinese BPES family, and the new candidate pathogenic genes and mutations were identified.

## 2 Materials and methods

### 2.1 Patients

In this study, a Chinese family with a history of BPES was recruited from the Plastic Surgery Hospital of Weifang Medical University. Of these nine family members, five were BPES^+^, of which four were female, and one was male. All of the family members denied consanguinity, improper drug use, had no history of disease or history of preterm birth.


**Informed consent:** Informed consent has been obtained from all individuals included in this study.
**Ethical approval:** The research related to human use has been complied with all the relevant national regulations, institutional policies, and in accordance with the tenets of the Helsinki Declaration and has been approved by the authors’ institutional review board or equivalent committee and was approved by the Ethics Committee of the Plastic Surgery Hospital of Weifang Medical University.

### 2.2 *FOXL2* gene mutation screening

Peripheral venous blood from BPES patients and healthy subjects was collected for genomic DNA extraction using TIANamp Genomic DNA Kit (TIANGEN BIOTECH, Beijing, China). The coding region of *FOXL2* was amplified by polymerase chain reaction (PCR) (forward primers: GAGTACCGGCAGATTTCAAG, GTTCGAGAAGGGCAACTACC, and CCTGACCTCTGTGACCTTGC. Reverse primers: AGTTGTTGAGGAAGCCAGAC, TGAGAGAGAGAGGCCAAGAGGTC, and AACAAAGCAGCAGCAGCGACAGC). Purified PCR products were sent to Beijing Liuhe BGI Technology Co., Ltd for sequencing. Lasergene software (DNASTAR, Inc., Madison, Wisconsin, USA) was used for the sequence alignment to screen for gene mutations. The wild-type *FOXL2* gene sequence was obtained from the National Center for Biotechnology Information’s (NCBI) website (https://www.ncbi.nlm.nih.gov/) [16].

### 2.3 WES

WES was performed by Beijing Novogene Biotech Co., Ltd. All BPES^+^ patients and the father (healthy subject) in this family participated in the study.

### 2.4 Data analysis

#### 2.4.1 Detection and screening of single-nucleotide variants (SNVs) and insertions/deletions (indels) of bases

Raw high-throughput sequencing data were evaluated for quality in the presence of a reference sequence or a reference genome (GRCh37/hg19). Filtering on raw reads was based on the sequencing error rate, the data volume, and the degree of similarity to obtain clean reads, which was used for downstream analysis. The resulting sequence data were compared to the reference genome using the Burrows-Wheeler Aligner (https://sourceforge.net/projects/bio-bwa/), and the comparison results were obtained [17]. SAMtools (http://samtools.sourceforge.net/) was used to sort the comparison results [18], and duplicate reads were identified and labeled using Picard Tools (https://sourceforge.net/projects/picard/). We then used the comparison results after repeated labeling to carry out the final statistical calculations.

#### 2.4.2 Filtering and screening of test results

**Figure 1:**
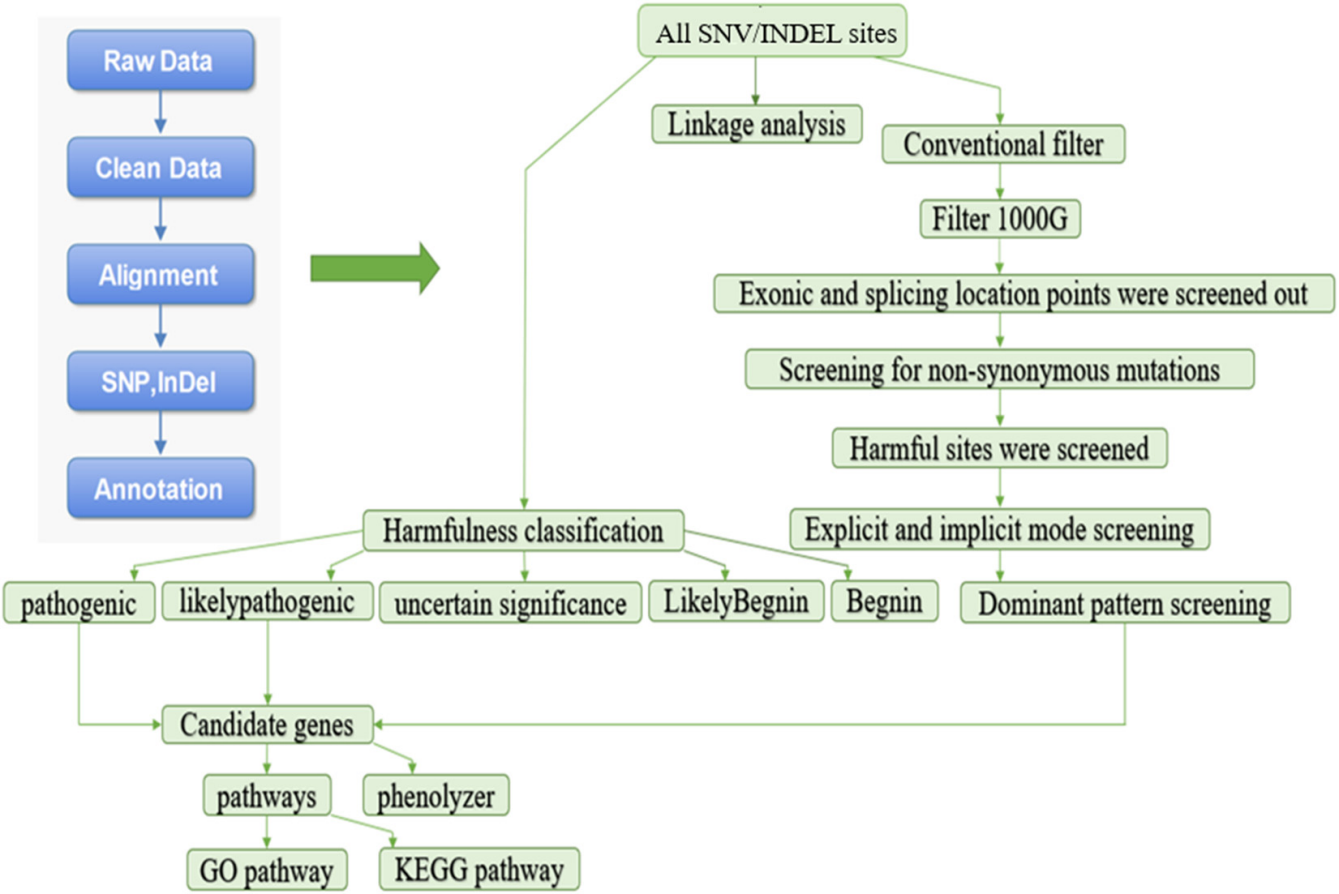
Flow chart of data analysis. Look in the direction indicated by the arrow.

Data were analyzed using NCBI dbSNP database, the 1000 Genomes Project, and other existing databases [19,20]. For the comparison results, we combined SIFT (http://sift.jcvi.org/), MutationTaster (https://www.mutationtaster.org/), Polyphen2 (http://gegetics.bwh.harvard.edu/pph2/), and other pathogenicity detection software to identify SNVs and indels using SAMTools and filtered the SNVs and indels by using the international filtering standards. ANNOVAR software (https://annovar.openbioinformatics.org/en/latest/) was used to annotate SNV and indel sites [21]. This mainly included four steps: screening based on mutation harmfulness, screening based on sample conditions, screening based on the candidate genes and their relationship with disease phenotypes, and finally obtaining the candidate pathogenic mutations (Figure 1).

#### 2.4.3 Linkage analysis

For linkage analysis, we used the Merlin tool (http://csg.sph.umich.edu/abecasis/Merlin/index.html) to combine the high-throughput sequencing data in the family with the allele frequency of the Chinese population in the HapMap database and used known SNVs as linkage markers to obtain the linkage candidate regions.

#### 2.4.4 Gene-disease phenotype analysis

We conducted significant enrichment analysis on the candidate genes, including gene ontology analysis and Kyoto Encyclopedia of Genes and Genomes (KEGG) pathway enrichment analysis. In this process, we referred to the theories and methods of Chen et al. [22], Yuan et al. [23], and Liu et al. [24], to identify the main metabolic and signal transduction pathways involved in these genes, analyzed the necessity of the candidate genes, and explored their relationship with the disease. Finally, we screened the candidate genes and ranked them according to how strongly they were associated with the disease.

## 3 Results

### 3.1 Clinical data analysis

**Figure 2:**
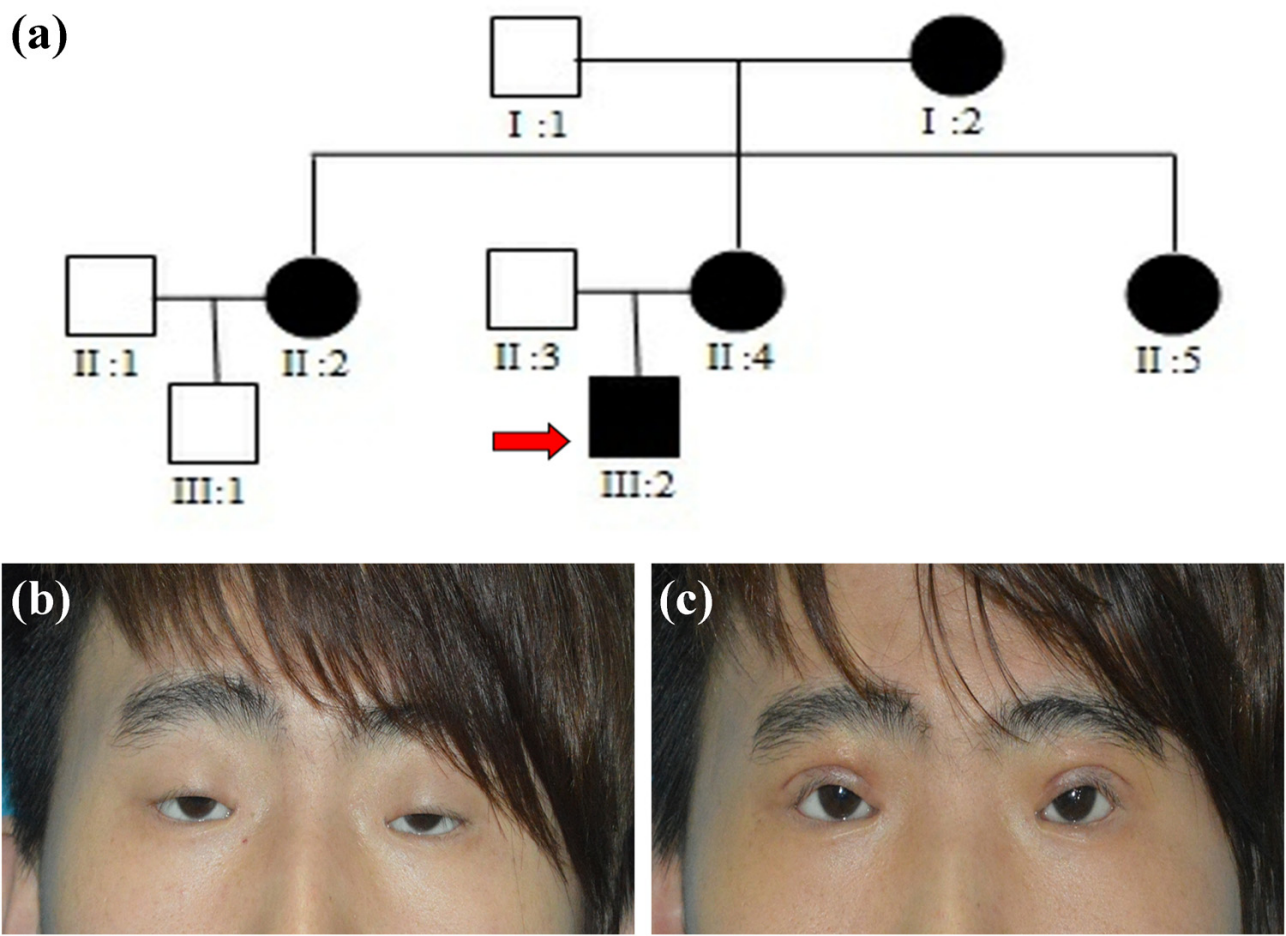
(a) The pedigrees of the Chinese BPES family, with the red arrows pointing to the proband of the family. (b) Preoperative photos of the proband. (c) Postoperative photo of the proband (7 days after surgery).

Among the nine family members included in this study, there were five with BPES (Figure 2a). The clinical information of the patients was obtained by three plastic surgeons and an ophthalmologist at the Plastic Surgery Hospital of Weifang Medical University (Table 1). All patients had the typical ocular features of BPES: narrow fissure, ptosis, inverted epicanthus, widened epicanthus spacing, and low and flat nose bridge. In addition, the family proband (III:2) was diagnosed with bilateral amblyopia, strabismus, ametropia, and ophthalmoplegia. Currently, there is no unified standard for the treatment of patients with BPES [25]. The family proband received frontal muscle fascial flap suspension surgery in our hospital. The operation was successful, and the patient’s prognosis was favorable (Figure 2b and c). We found that except for one female patient (II:5 – young and unmarried with regular menstrual history, but had never given birth), the other females had normal menstruation and fertility, confirming that these patients have type II BPES.

**Table 1:** Clinical data of the patients

Patients	Age (years)	IICD (mm)	HPFL (mm)		IPFH (mm)		Levator function (mm)	
			LE	RE	LE	RE	LE	RE
I:2	64	36	25	25	4	3	4	4
II:2	43	37	23	24	4	4	2	2
II:4	42	38	22	22	5	5	0	0
II:5	40	36	23	22	5	4	2	2
III:2	18	40	22	25	5	6	0	0

Abbreviations: IICD, inner intercanthal distance; HPFL, horizontal palpebral fissure length; IPFH, vertical interpalpebral fissure height; LE, left eye; and RE, right eye.

### 3.2 Candidate pathogenic variants in patients with BPES

According to Sanger sequencing analysis, the *FOXL2* gene mutation screening results of the BPES^+^ patients recruited in this study were all negative. After WES analysis, we screened out a total of 52,215 mutation sites, detected 1,211 harmful mutations that might affect protein function, and obtained 33 pathogenic variants (SNV variants) and 11 likely pathogenic variants (including five SNV variants and six indel variants) through harmful degree analysis. A total of 44 variants and 43 candidate genes were identified (two SNV mutation sites were detected on the pathogenic candidate gene *FLG* [Filaggrin]). We also detected 20 candidate genes corresponding to SNVs and one candidate gene corresponding to indels through dominant inheritance pattern screening. There were 21 candidate genes and 21 mutation sites. Among them, the candidate gene *FLG* corresponding to an SNV overlapped with the harmfulness detection results, but the mutation sites were different. In the end, we obtained 63 candidate genes with 65 mutation sites (three mutation sites were detected on the candidate gene *FLG*), among which 58 SNV sites and 7 indel sites were identified.

### 3.3 Linkage analysis results

**Figure 3:**
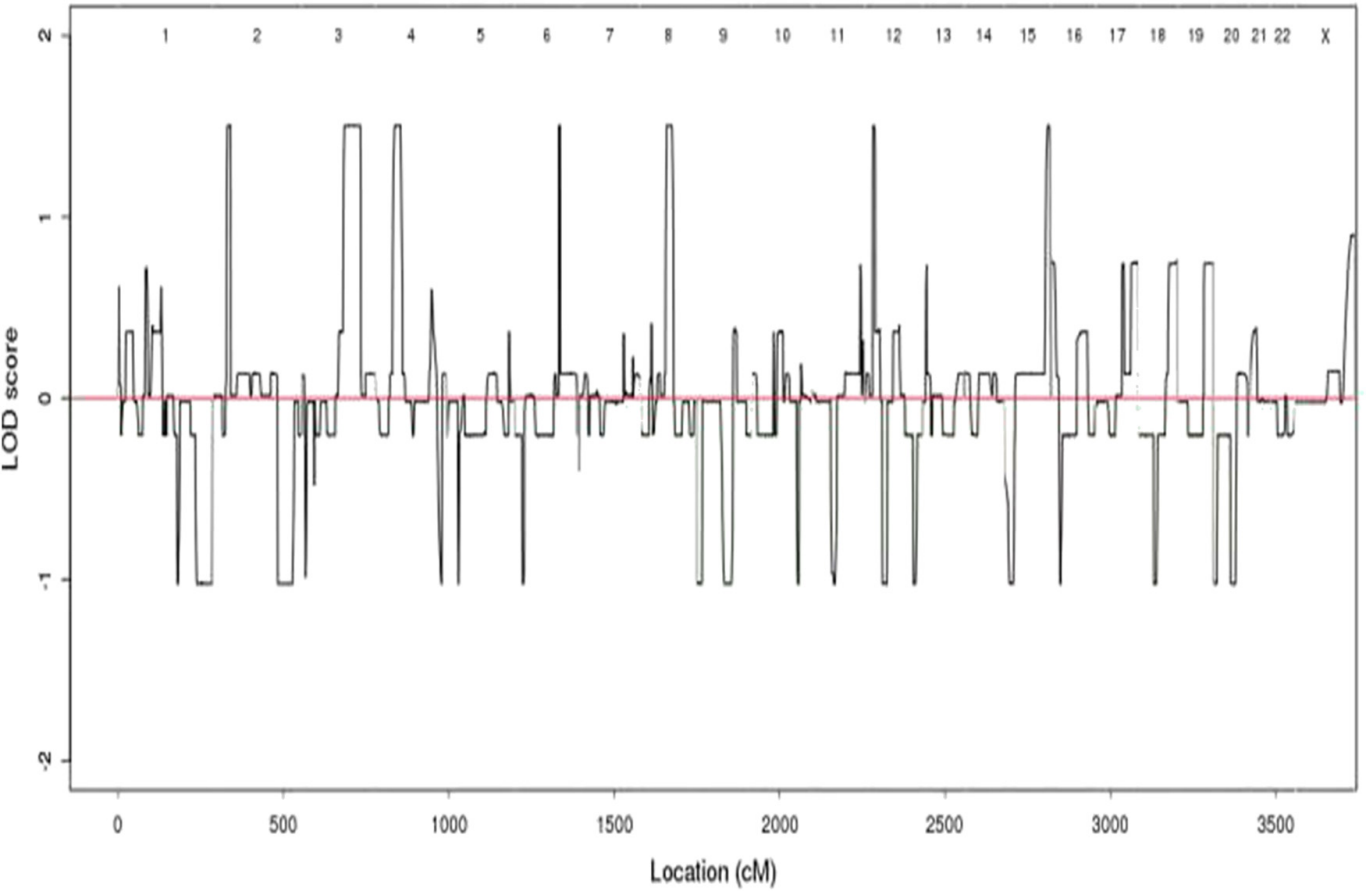
Diagram of linkage analysis results. The *X*-axis represents the genetic distance, represented in centimos (cM). The *Y*-axis represents the LOD value.

The linkage analysis results showed that 27, 120, 46, 14, 49, 21, and 17 SNVs were obtained on chromosomes 2, 3, 4, 6, 8, 12, and 15, respectively. The log odds score (LOD) values of these SNVs were greater than 1.5 (Figure 3). However, LOD values of all results, although greater than 1 but less than 3, indicate possible linkage, indicating that mutations at these sites may have cosegregation of disease phenotypes.

### 3.4 Screening based on candidate genes and their relationship with disease phenotypes

**Figure 4:**
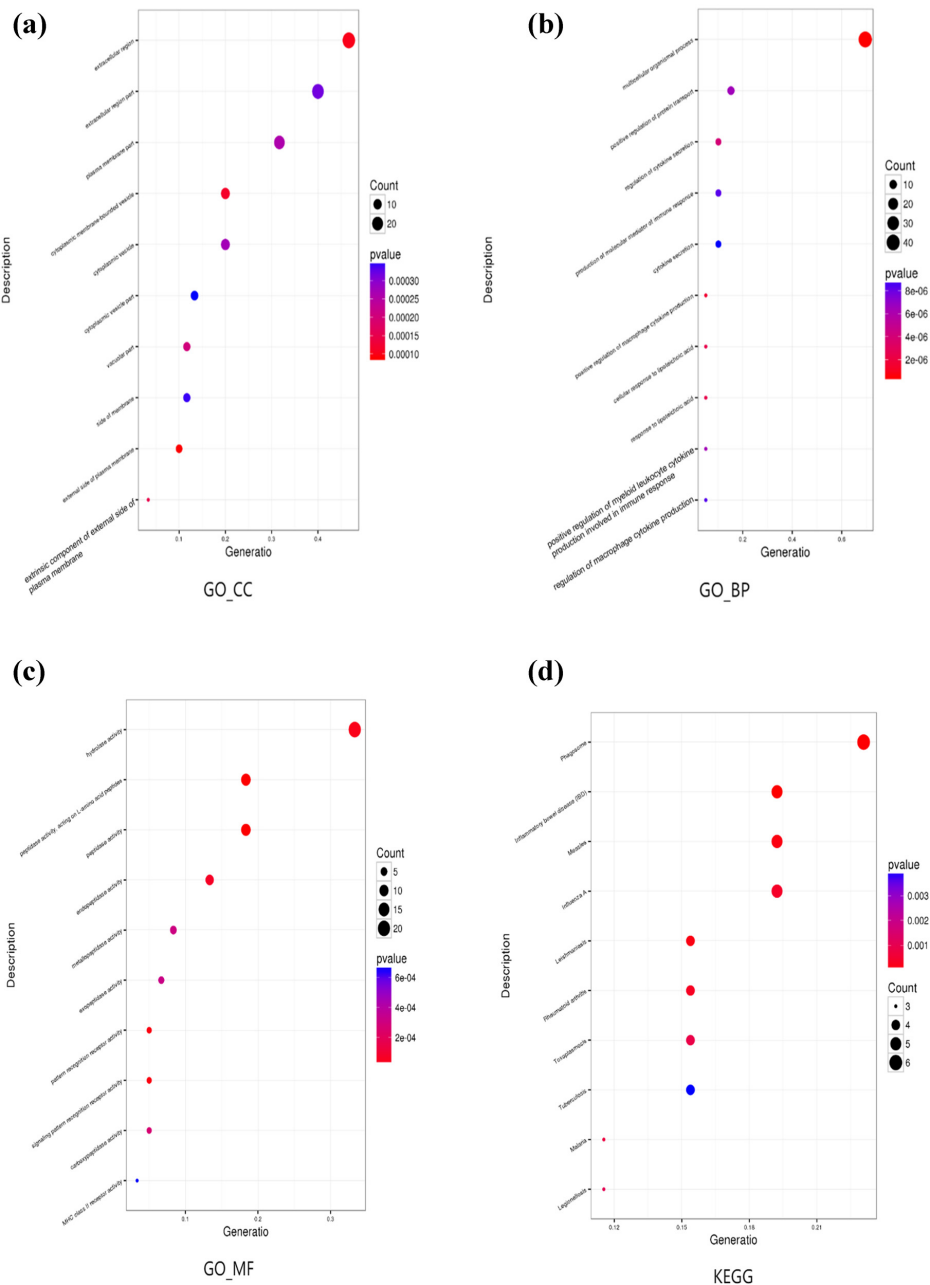
Scatter diagram of (a) gene ontology cell component (GO_CC) functional enrichment; (b) gene ontology biological pathway (GO_BP) functional enrichment; (c) gene ontology molecular function (GO_MF) functional enrichment; and (d) KEGG pathway enrichment.

**Figure 5:**
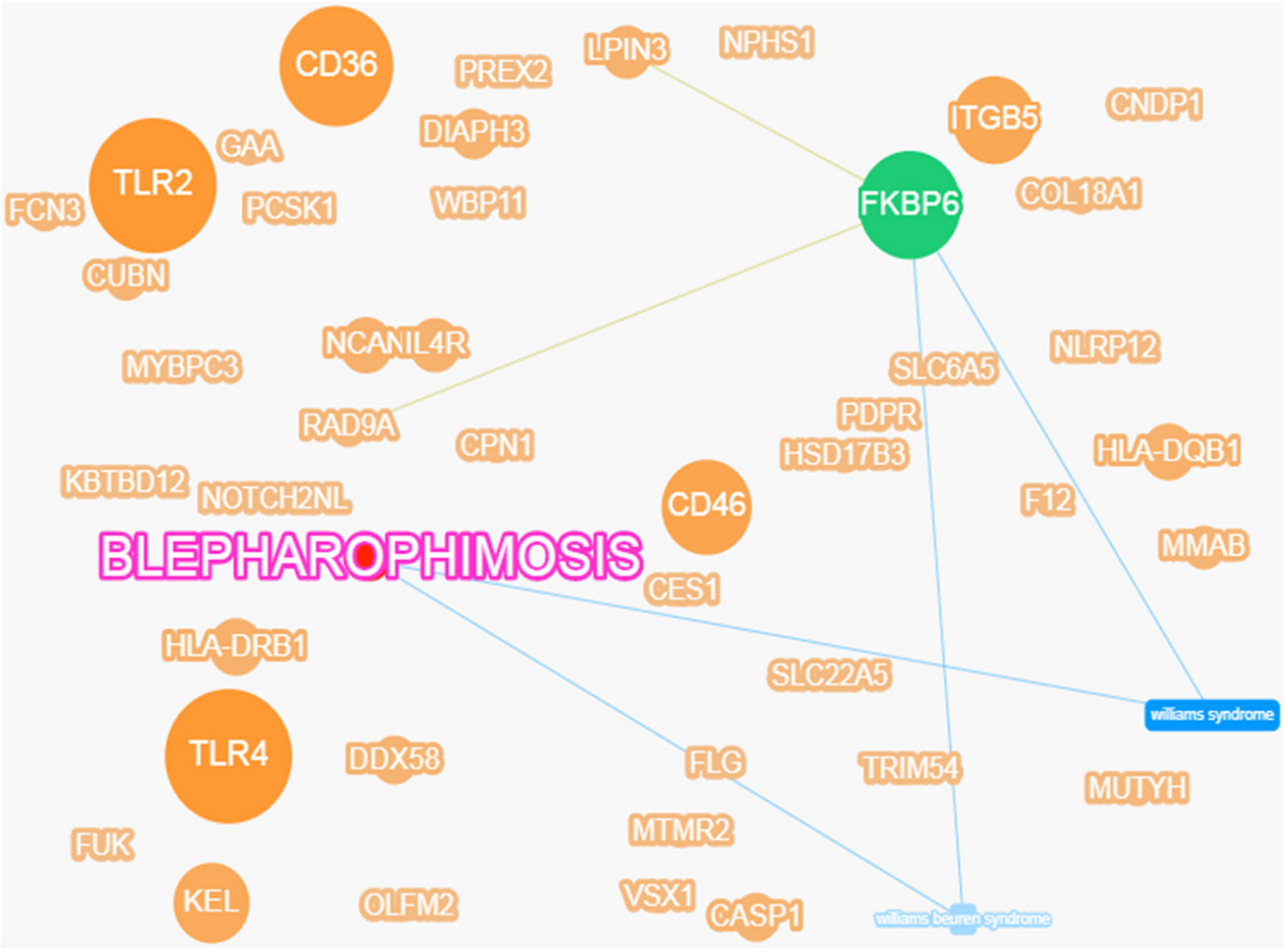
Gene-disease phenotype association network diagram. The size of the spots indicates the strength of the gene’s association with disease. The larger the dot, the stronger the correlation. The green dot indicates genes that have been reported or found to be associated with the related disease in the database. The lines indicate that the genes or diseases on either side of the line are related.

Gene ontology functional enrichment analysis could generate three results: cell component, biological pathway, and molecular function. Among them, 60 variants were enriched in cell component, 59 in biological pathway, and 60 in molecular function (Figure 4a–c). Enrichment analysis of KEGG pathway revealed seven variants (Figure 4d). At the same time, we screened candidate genes and ranked them according to how strongly they were associated with the disease (Figure 5). The top seven genes were toll-like receptor (*TLR*) *2*, *TLR4*, *CD36*, FKBP prolyl isomerase family member 6, inactive (*FKBP6*), *CD46*, *ITGB5*, and Kell metallo-endopeptidase (kell blood group, *KEL*). The variants corresponding to these candidate genes are all SNVs. According to the American Society of Medical Genetics and Genomics (ACMG) variation classification standard, *ITGB5* currently has no disease-causing grade, whereas the remaining genes do, and their genetic mode is heterozygous. Of these, *ITGB5* was the strongest gene associated with the disease among the genes screened by the dominant pattern. *TLR2* showed the strongest association with disease. However, this mutation did not come from the proband maternal lineage but from the proband father (the healthy subject). This indicates that the mutation of this gene is not directly related to the incidence of BPES. In addition, there may be other underlying diseases in the proband and his father that have not been screened for. *FKBP6* is associated with the Lipin 3 (*LPIN3*) gene and the RAD9 checkpoint clamp component A (*RAD9A*) gene and has been associated with Williams syndrome and Williams-Beuren syndrome, but *FKBP6* does not have an advantage in terms of the strength of association with the target disease. We annotated the top seven genes based on the screened candidate genes (Table 2).

**Table 2:** The information of the top seven genes

Gene	Location on a chromosome	The information of variants	Reference gene	Sample gene	Mutations	The number of samples	GO_CC	GO_BP	GO_MF	KEGG
*TLR2*	4q31.3	Heterozygous/exon/pathogenic	C	T	c.1339C>T	II:3/III:2	+	+	+	+
*TLR4*	9q33.1	Heterozygous/exon/pathogenic	G	A	c.820G>A, etc	II:3/III:2	+	+	−	+
*CD36*	7q21.11	Heterozygous/exon/pathogenic	C	T	c.1039C>T, etc	II:2/II:4	+	+	−	+
*FKBP6*	7q11.23	Heterozygous/exon/likely pathogenic	C	T	c.201C>T, etc	I:2/II:5	−	+	+	−
*CD46*	1q32.2	Heterozygous/exon/pathogenic	C	T	c.38C>T, etc	II:4	+	−	+	+
*ITGB5*	3q21.2	Heterozygous/exon/dominant inheritance	A	G	c.608T>C	I:2/II:2/II:4/II:5/III:2	−	−	−	+
*KEL*	7q34	Heterozygous/exon/pathogenic	T	A	c.1481A>T	II:2,II:4,III:2	+	+	+	−

“+”: the gene is expressed in this pathway.

“–”: the gene is not expressed in this pathway.

### 3.5 Prediction and analysis of *ITGB5*

**Figure 6:**
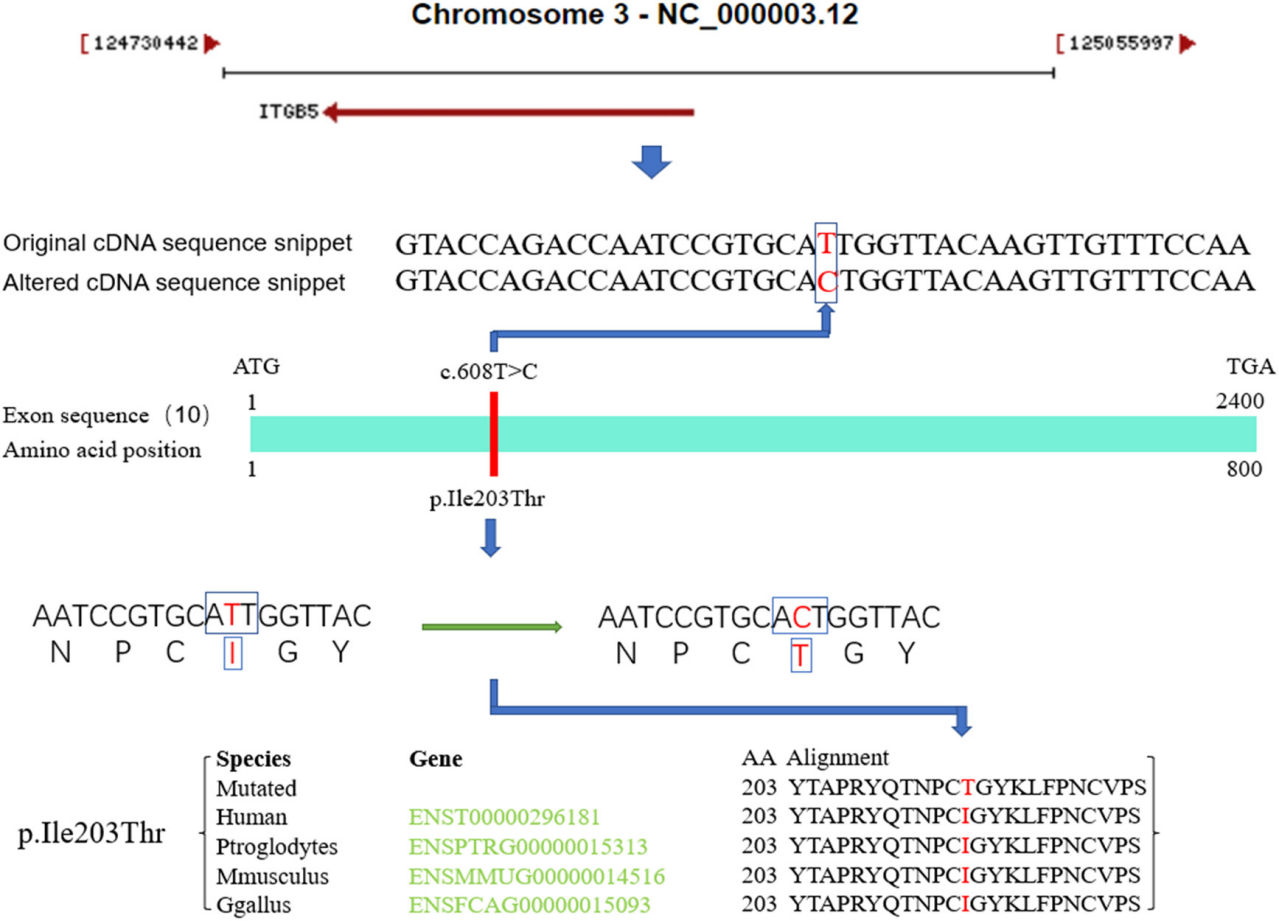
Schematic of the *ITGB5* mutation site, which is located on the 10th exon of *ITGB5* and highly conserved across species. Main sources of data information: NCBI (https://www.ncbi.nlm.nih.gov/), MutationTaster (http://www.mutationtaster.org/).

The *ITGB5* gene, which encodes the β subunit of integrin and can combine with different α chains to form a variety of heterodimers of integrin, is the strongest gene associated with the disease among the genes screened by the dominant pattern. Integrins are complete cell surface receptors involved in cell adhesion and cell surface-mediated signal transduction. α-v, β-5 integrins are involved in the adhesion of the vitreous. This gene is also involved in the pathogenesis of keloids, diarrhea, glioblastomas, pancreatic cancer, breast cancer, and liver cancer, among other diseases [26,27,28,29,30,31]. We performed the data analysis based on NCBI website, pathogenicity prediction software, and research data (Figure 6). A missense mutation (c.608T>C, p.Ile203Thr) in the *ITGB5* gene can change isoleucine at the 203rd position of the amino acid sequence to threonine, causing changes in the splicing sites and protein function. According to the analysis of amino acid conservatism, this mutation is highly conserved across species. Moreover, the LOD value of this mutation is greater than 1.5, suggesting the possibility of cosegregation of disease phenotypes. This mutation was present in exons of all BPES patients in this family, suggesting that this gene mutation may be related to the pathogenesis of BPES. In addition, the *ITGB5* gene is located on the long arm of chromosome 3 (3q21.2), which is close to *FOXL2* (3q23), a known pathogenic gene of BPES, and located upstream of the *FOXL2* gene.

## 4 Discussion

In this study, we recruited a Chinese BPES^+^ family with facial features typical of BPES, and through analysis of clinical data from the female members, this pedigree was confirmed to be type II BPES. We conducted *FOXL2* gene screening on DNA samples from family members and found no *FOXL2* gene mutation in this family, indicating that *FOXL2* is not the pathogenic gene in this BPES family. Subsequently, WES was performed to screen for SNVs and indels [32]. There are 3.6–4.4 M single-nucleotide polymorphisms (SNPs) in the human genome, and the vast majority (more than 95%) of high-frequency SNPs (population medium gene frequency >5%) are recorded in the dbSNP database, and the high-frequency SNPs are generally not the main mutation sites for disease [33]. At the same time, the human genome has about 350k indels at coding regions or splicing sites that may alter the translation of proteins. The results of mutation tests are usually enormous, but the number of mutations that are truly relevant to the disease being studied is limited. To screen for mutations truly related to the disease under study from the massive mutation-detection results, we need to conduct further analysis and screening of this data.

In 2015, the ACMG developed standards and guidelines for the interpretation of sequence variation, which became the gold standard for the interpretation of data after high-throughput sequencing [34]. The ACMG has developed a variation classification system and recommends specific standard terminology, which divides the variation into five types, including pathogenic, likely pathogenic, uncertain significance, likely benign, and benign, to describe mutations found in genes responsible for Mendelian diseases. Based on the existing information analysis methods, we classified the harmfulness of mutation sites and obtained 33 pathogenic variants and 11 likely pathogenic variants. Subsequently, we screened for dominant inheritance patterns of Mendelian diseases. In dominant diseases, the pathogenic mutation from either parent is usually heterozygous, and the heterozygous mutation on the candidate gene should be considered first. The candidate sites we choose should be heterozygous mutations on the autosomes of patients but not on the chromosomes of healthy individuals. The BPES family in this study had three successive generations of onset, and the clinical phenotype was typical and single. Both male and female family members had the disease, which was in line with an autosomal-dominant inheritance pattern, and the disease showed a trend of aggravation by generation. We detected 20 candidate genes corresponding to SNVs and one candidate gene corresponding to an indel in this family. Subsequently, we identified the most important biochemical metabolic pathways and signal transduction pathways involved by the mutant genes through significant enrichment analysis and screened out the genes strongly associated with the disease through genotypic phenotype 1 analysis. In the end, we found that *ITGB5* was the gene that was most strongly associated with disease in the genes screened by dominant pattern.

The human *ITGB5* gene is a member of the integrin family, and its main function is to participate in the adhesion of immune cells. *ITGB5* is commonly present as a dimer, including α and β subunits, which preferentially bind to cellular adhesion molecules and constitute components of the extracellular matrix. *ITGB5* is highly expressed in the lung and moderately expressed in the spleen and shows low expression in the small intestine, lymph, thymus, and liver. This gene has a variety of functions in biological processes, including cell migration during cell growth and wound repair, cell variation and apoptosis, and regulation of potential metastasis of some tumor cells.

A mutation in the *ITGB5* gene (c.608T>C, p.Ile203Thr) appeared in the base sequence of all patients with BPES in this family. Based on linkage analysis, the LOD value of this mutation was greater than 1.5, suggesting the possibility of cosegregation of disease phenotypes. In addition, *ITGB5* is located on the long arm of chromosome 3 (3q21.2) and is just upstream of the known pathogenic gene of BPES, *FOXL2* (3q23). We speculate that it may be directly involved in the pathogenesis of BPES or indirectly involved through the regulation of *FOXL2*.

## 5 Conclusion

In summary, through our in-depth screening and analytical methods, we detected a possible pathogenic gene mutation in a typical Chinese BPES family, other than the *FOXL2* gene mutation. Using WES, we discovered this new candidate pathogenic gene to be *ITGB5*. This study is the first to describe that a mutation in *ITGB5* could lead to genetic pathogenesis of BPES, and further studies with a larger patient cohort are needed to verify this novel finding.

## References

[1] Beaconsfield M, Walker JW, Collin JR. Visual development in the blepharophimosis syndrome. Br J Ophthalmol. 1991;75(12):746–8. 10.1136/bjo.75.12.746 PMC1042558 1768667

[2] Chawla B, Bhadange Y, Dada R, Kumar M, Sharma S, Bajaj MS, et al. Clinical, radiologic, and genetic features in blepharophimosis, ptosis, and epicanthus inversus syndrome in the Indian population. Invest Ophthalmol Vis Sci. 2013;54(4):2985–91. 10.1167/iovs.13-11794 23513057

[3] Ishikiriyama S, Goto M. Blepharophimosis, ptosis, and epicanthus inversus syndrome (BPES) and microcephaly. Am J Med Genet. 1994;52(2):245. 10.1002/ajmg.1320520228 7802022

[4] Castets S, Roucher-Boulez F, Saveanu A, Mallet-Motak D, Chabre O, Amati-Bonneau P, et al. Hypopituitarism in patients with blepharophimosis and FOXL2 mutations. Horm Res Paediatr. 2020;93(1):30–9. 10.1159/000507249 32454486

[5] Treier M, Gleiberman AS, O'connell SM, Szeto DP, McMahon JA, McMahon AP, et al. Multistep signaling requirements for pituitary organogenesis in vivo. Genes Dev. 1998;12(11):1691–704. 10.1101/gad.12.11.1691 PMC316866 9620855

[6] de Ru MH, Gille JJ, Nieuwint AW, Bijlsma JB, van der Blij JF, van Hagen JM. Interstitial deletion in 3q in a patient with blepharophimosis-ptosis-epicanthus inversus syndrome (BPES) and microcephaly, mild mental retardation and growth delay: clinical report and review of the literature. Am J Med Genet A. 2005;137(1):81–7. 10.1002/ajmg.a.30786 16015581

[7] Jamshidian-Tehrani M, Cheraqpour K, Nezamslami A. Association between blepharophimosis-ptosis-epicanthus inversus syndrome and lacrimal system anomalies. Orbit. 2021;23:1–5. 10.1080/01676830.2021.1980892 34555988

[8] Méjécase C, Nigam C, Moosajee M, Bladen JC. The genetic and clinical features of FOXL2-related blepharophimosis, ptosis and epicanthus inversus syndrome. Genes (Basel). 2021;12(3):364. 10.3390/genes12030364 PMC7998575 33806295

[9] Jiang J, Yuan H, Zheng X, Wang Q, Kuang T, Li J, et al. Gene markers for exon capture and phylogenomics in ray-finned fishes. Ecol Evol. 2019;9(7):3973–83. 10.1002/ece3.5026 PMC6468074 31015981

[10] Rasool IG, Zahoor MY, Iqbal M, Anjum AA, Ashraf F, Abbas HQ, et al. Whole exome sequencing revealed novel variants in consanguineous Pakistani families with intellectual disability. Genes Genomics. 2021;43(5):503–12. 10.1007/s13258-021-01070-7 33710595

[11] Mustafa S, Akhtar Z, Latif M, Hassan M, Faisal M, Iqbal F. A novel nonsense mutation in NPR2 gene causing Acromesomelic dysplasia, type Maroteaux in a consanguineous family in Southern Punjab (Pakistan). Genes Genomics. 2020;42(8):847–54. 10.1007/s13258-020-00955-3 PMC7374443 32506268

[12] Shendure J, Ji H. Next-generation DNA sequencing. Nat Biotechnol. 2008;26(10):1135–45. 10.1038/nbt1486 18846087

[13] Li F, Chai P, Fan J, Wang X, Lu W, Li J, et al. A novel FOXL2 mutation implying blepharophimosis-ptosis-epicanthus inversus syndrome Type I. Cell Physiol Biochem. 2018;45(1):203–11. 10.1159/000486358 29339661

[14] Hu J, Ke H, Luo W, Yang Y, Liu H, Li G, et al. A novel FOXL2 mutation in two infertile patients with blepharophimosis-ptosis-epicanthus inversus syndrome. J Assist Reprod Genet. 2020;37(1):223–9. 10.1007/s10815-019-01651-2 PMC7000634 31823134

[15] Han Y, Wu J, Yang W, Wang D, Zhang T, Cheng M. New STAT3-FOXL2 pathway and its function in cancer cells. BMC. Mol Cell Biol. 2019;20(1):17. 10.1186/s12860-019-0206-3 PMC6587274 31221094

[16] Sayers EW, Beck J, Bolton EE, Bourexis D, Brister JR, Canese K, et al. Database resources of the National center for biotechnology information. Nucleic Acids Res. 2021;49(D1):D10–7. 10.1093/nar/gkaa892 PMC7778943 33095870

[17] Li H, Durbin R. Fast and accurate short read alignment with Burrows–Wheeler transform. Bioinformatics. 2009;25(14):1754–60. 10.1093/bioinformatics/btp324 PMC2705234 19451168

[18] Li H, Handsaker B, Wysoker A, Fennell T, Ruan J, Homer N, et al. The sequence alignment/map format and SAMtools. Bioinformatics. 2009;25(16):2078–9. 10.1093/bioinformatics/btp352 PMC2723002 19505943

[19] Hindorff LA, Sethupathy P, Junkins HA, Ramos EM, Mehta JP, Collins FS, et al. Potential etiologic and functional implications of genome-wide association loci for human diseases and traits. Proc Natl Acad Sci U S A. 2009;106(23):9362–7. 10.1073/pnas.0903103106 PMC2687147 19474294

[20] Welter D, MacArthur J, Morales J, Burdett T, Hall P, Junkins H, et al. The NHGRI GWAS catalog, a curated resource of SNP-trait associations. Nucleic Acids Res. 2014;42(Database issue):D1001–6. 10.1093/nar/gkt1229 PMC3965119 24316577

[21] Wang K, Li M, Hakonarson H. ANNOVAR: functional annotation of genetic variants from high-throughput sequencing data. Nucleic Acids Res. 2010;38(16):e164. 10.1093/nar/gkq603 PMC2938201 20601685

[22] Chen L, Zhang YH, Wang S, Zhang Y, Huang T, Cai YD. Prediction and analysis of essential genes using the enrichments of gene ontology and KEGG pathways. PLoS One. 2017;12(9):e0184129. 10.1371/journal.pone.0184129 PMC5584762 28873455

[23] Yuan HY, Lv YJ, Chen Y, Li D, Li X, Qu J, et al. TEAD4 is a novel independent predictor of prognosis in LGG patients with IDH mutation. Open Life Sci. 2021;16(1):323–35. 10.1515/biol-2021-0039 PMC8042920 33889755

[24] Liu B, Shi H, Qiu W, Wu X, Li L, Wu W. A two-microRNA signature predicts the progression of male thyroid cancer. Open Life Sci. 2021;16(1):981–91. 10.1515/biol-2021-0099 PMC8439266 34595349

[25] Ben Abdesslem N, Knani L, Mili W, Mahjoub A, Ben Rayana N, Ghorbel M, et al. Prise en charge du syndrome de blépharophimosis ptôsis épicanthus inversus (SBPE) dans un centre de référence en Tunisie [Management of blepharophimosis, ptosis, epicanthus inversus syndrome at a referral center in Tunisia]. J Fr Ophtalmol. 2021;44(1):53–62. 10.1016/j.jfo.2020.03.021 33279286

[26] Yang T, Chen M, Ma K, Fu X. The effects of down-regulated ITGB5 expression on the proliferation of keloid fibroblasts. Zhonghua Zheng Xing Wai Ke Za Zhi. 2017;33(1):49–52. 30070797

[27] Zhou C, Liu Z, Liu Y, Fu W, Ding X, Liu J, et al. Gene silencing of porcine MUC13 and ITGB5: candidate genes towards Escherichia coli F4ac adhesion. PLoS One. 2013;8(7):e70303. 10.1371/journal.pone.0070303 PMC3726385 23922972

[28] Zhang LY, Guo Q, Guan GF, Cheng W, Cheng P, Wu AH. Integrin beta 5 is a prognostic biomarker and potential therapeutic target in Glioblastoma. Front Oncol. 2019;9:904. 10.3389/fonc.2019.00904 PMC6764112 31616629

[29] Zhuang H, Zhou Z, Ma Z, Li Z, Liu C, Huang S, et al. Characterization of the prognostic and oncologic values of ITGB superfamily members in pancreatic cancer. J Cell Mol Med. 2020;24(22):13481–93. 10.1111/jcmm.15990 PMC7701563 33073486

[30] Gu Y, Liu Y, Fu L, Zhai L, Zhu J, Han Y, et al. Tumor-educated B cells selectively promote breast cancer lymph node metastasis by HSPA4-targeting IgG. Nat Med. 2019;25(2):312–22. 10.1038/s41591-018-0309-y 30643287

[31] Lin Z, He R, Luo H, Lu C, Ning Z, Wu Y, et al. Integrin-β5, a miR-185-targeted gene, promotes hepatocellular carcinoma tumorigenesis by regulating β-catenin stability. J Exp Clin Cancer Res. 2018;37(1):17. 10.1186/s13046-018-0691-9 PMC5793391 29386044

[32] Neininger K, Marschall T, Helms V. SNP and indel frequencies at transcription start sites and at canonical and alternative translation initiation sites in the human genome. PLoS One. 2019;14(4):e0214816. 10.1371/journal.pone.0214816 PMC6461226 30978217

[33] Sherry ST, Ward MH, Kholodov M, Baker J, Phan L, Smigielski EM, et al. dbSNP: the NCBI database of genetic variation. Nucleic Acids Res. 2001;29(1):308–11. 10.1093/nar/29.1.308 PMC29783 11125122

[34] Richards S, Aziz N, Bale S, Bick D, Das S, Gastier-Foster J, et al. Standards and guidelines for the interpretation of sequence variants: a joint consensus recommendation of the American college of medical genetics and genomics and the association for molecular pathology. Genet Med. 2015;17(5):405–24. 10.1038/gim.2015.30 PMC4544753 25741868

